# Effects of Preanhepatic Glucose-Insulin Infusion on Metabolic and Hemodynamic Outcomes in Liver Transplantation: A Randomized Clinical Trial: Preanhepatic Glucose-Insulin Infusion and Liver Transplantation

**DOI:** 10.31661/gmj.v14i.3971

**Published:** 2025-08-19

**Authors:** Mohammadreza Moshari, Mohammad Gharehbeglou, Shideh Dabir, Maryam Vosoughian, Mastaneh Dahi, Ali Dabbagh, Soudeh Tabashi, Mohsen Ariannik, Firoozeh Madadi, Jamshid Ordoni Avval

**Affiliations:** ^1^ Department of Anesthesiology, School of Medicine, Shahid Beheshti University of Medical Sciences, Tehran, Iran; ^2^ Musculoskeletal Injuries Research Center, Shahid Beheshti University of Medical Sciences, Tehran, Iran; ^3^ Anesthesiology Research Center, Shahid Beheshti University of Medical Sciences, Tehran, Iran; ^4^ Department of Anesthesiology and Critical Care Medicine, School of Medicine, Zahedan University of Medical Sciences, Zahedan, Iran

**Keywords:** Liver Transplantation, Glucose-Insulin Infusion, Hemodynamic Stability, Metabolic Outcomes, Randomized Controlled Trial

## Abstract

**Background:**

Liver transplantation is a complex procedure requiring meticulous perioperative management to optimize outcomes. The administration of glucose-insulin solutions during the preanhepatic phase may influence hemodynamic and metabolic stability. This study aimed to evaluate the effects of dextrose-insulin infusion on hemodynamic, metabolic, and clinical outcomes in liver transplant recipients.

**Materials and Methods:**

A randomized, double-blinded clinical trial was conducted on 88 patients (1:1 allocation) undergoing liver transplantation at Taleghani Hospital, Iran. The intervention group received dextrose 50% (1g/kg) and insulin (1u/kg), while the control group received normal saline. Hemodynamic parameters (cardiac output, vascular resistance), metabolic markers (glucose, potassium, lactate), and clinical outcomes (hospital stay, complications) were assessed across preanhepatic, anhepatic, and reperfusion phases. Statistical analysis was performed using SPSS v27.0.

**Results:**

The intervention group demonstrated significantly higher cardiac output (6.95±1.66 vs. 5.56±2.02 L/min, P0.001) and lower inotropic requirements (65.9% vs. 88.6%, P=0.018) during the anhepatic phase. Postoperatively, the intervention group had reduced ALT (P=0.038), AST (P=0.019), bilirubin (P=0.021), BNP (P=0.002), and lactate (P0.001). No differences were observed in hospital stay, ICU duration, or complication rates.

**Conclusion:**

Dextrose-insulin administration during liver transplantation improves intraoperative hemodynamics and reduces postoperative metabolic stress without increasing adverse events.

## Introduction

Liver transplantation is a life-saving procedure for patients with end-stage liver disease caused by various underlying conditions, including viral hepatitis, alcoholic liver disease, metabolic disorders, and hepatocellular carcinoma [1]. Liver transplantation is a complex surgical procedure divided into three critical phases, preanhepatic, anhepatic, and reperfusion. Each phase presents physiological and surgical challenges, significantly impacting patient outcomes [2].

The preanhepatic phase begins with the initial incision and ends with the complete vascular occlusion of the portal vein, inferior vena cava, and hepatic artery. This stage is characterized by major hemodynamic changes and significant blood loss due to the liver’s complex vascular anatomy, often requiring meticulous anesthetic and surgical management [3]. Coagulation disturbances, particularly hyperfibrinolysis, can further complicate intraoperative bleeding and persist into the anhepatic phase [4][5]. Additionally, systemic inflammatory response syndrome (SIRS) is a major concern during this phase, because prolonged surgery, extensive blood loss, and ischemia-reperfusion injury can trigger excessive activation of Toll-like receptors (TLR2/4), leading to systemic complications such as acute kidney injury (AKI)

[6][7].

Metabolic alterations during the preanhepatic phase, particularly in glucose metabolism and insulin sensitivity, play a crucial role in post-transplant outcomes. Intraoperative hyperglycemia is a common complication due to surgical stress, transfusion requirements, and corticosteroid use [8]. Insulin therapy has been suggested as a key intervention to control intraoperative glucose levels, effectively reducing hyperglycemia-related complications [9] and also improving postoperative liver function, preserving hepatic glycogen stores and providing anti-apoptotic and anti-inflammatory effects during the perioperative period [10]. The metabolic effects of liver transplantation extend beyond the immediate postoperative period because patients frequently experience long-term disruptions in glucose metabolism and insulin sensitivity, complicating their management [11].

The efficacy and safety of insulin-dextrose regimens in liver transplantation and major hepatic resections remain contentious, with studies yielding conflicting results [9][10][12][13][14][15]. Gedik et al. [13] found that dextrose infusion alone (without insulin) maintained safer blood glucose levels compared to combined insulin-dextrose therapy in living-donor liver transplant recipients, suggesting that exogenous insulin may provoke hyperglycemia, particularly during the neohepatic phase. Conversely, Omiya et al. (2022) demonstrated that hyperinsulinemic-normoglycemic clamp (HNC) therapy during liver resections significantly reduced surgical site infections (SSIs), though it did not improve postoperative hepatic function, possibly due to the absence of preoperative carbohydrate loading, which may be critical for hepatic glycogen preservation [10]. Sato et al. [14] and Kang et al. [9] further reinforced the benefits of insulin-glucose infusions, with the latter showing that Portland intensive insulin therapy (PoIIT) reduced post-reperfusion hyperglycemia and complications like biliary strictures and infections in liver transplant recipients. These findings align with Cywes et al. [15], who observed that intraportal glucose infusion enhanced glycogen stores and attenuated ischemic injury during transplantation. Critically, Hassanain et al. [9] reported that preoperative glycogen replenishment via insulin-dextrose protocols reduced postoperative liver dysfunction, emphasizing the role of hepatic glycogen as a protective energy reserve.

Targeted insulin therapy could be a practical approach for intraoperative metabolic control and may improve post-transplant outcomes. So, the present study aims to evaluate the effect of glucose-insulin infusion during the preanhepatic phase of liver transplantation on the metabolic and hemodynamic status of other phases of liver transplantation and overall patient outcomes after transplantation.

## Patients and Method

### Study design

The present study is a randomized, double-blinded clinical trial with a parallel group. The study protocol was approved by the Ethics Committee of the Faculty of Medicine, Shahid Beheshti University of Medical Sciences (ID: IR.SBMU.MSP.REC.1403.076). It was also approved in the clinical trial center registry —https://irct.behdasht.gov.ir/trial/69397 (code: IRCT20240606062025N1).

### Participants

From June 2024 to October 2024, candidate patients for liver transplantation aged 18 to 65 were included in this study. Patients with diabetes, potassium less than 3mmol/L, and patients who expired within the first 24 hours after transplantation were excluded from the study. Based on the study by Omiya et al. [12], considering infection rates within 30 days after surgery as the power for sample size calculation, this rate in control (31%) and hyperinsulinemic normoglycemia (HNC) (6%) groups were used to perform a sample size calculation with a dichotomous endpoint in a two-independent-sample study. Assuming an enrollment ratio of 1, a type I error rate (alpha) of 0.05, and 80% power (beta = 0.2), the initial estimated sample size was 37 participants per group (total N = 74) to detect a clinically meaningful difference in infection incidence. To account for potential participant attrition due to dropouts or exclusion criteria, an additional 20% was included, resulting in an adjusted target sample size of 45 per group (total N = 90).

### Randomization and intervention

The intervention was started after the treatment protocol was explained to the patients and written informed consent was obtained. Patients were randomly assigned to one of two treatment arms to ensure unbiased allocation using a block randomization method. The block size was four, accommodating three patients per block by the enrollment order. This randomization process was executed using the online block randomization software ‘Sealed Envelope,’ the randomized block lists were enclosed in sealed envelopes. The anesthesiologist opened a new envelope on the procedure day to determine the patient’s allocation.

All patients underwent standard monitoring throughout the Liver transplantation surgery, including electrocardiography (ECG), pulse oximetry, invasive blood pressure, and temperature monitoring. Anesthesia induction was achieved with fentanyl (2-5 μg/kg), propofol (1-2 mg/kg), lidocaine (1 mg/kg), and cisatracurium (0.2 mg/kg). For maintenance, isoflurane was administered to maintain the Bispectral Index (BIS) between 40 and 60. Vital signs were continuously monitored during surgery, with inotropic requirements recorded in the preanhepatic, anhepatic, and neoanhepatic phases. Two fixed, experienced surgeons carried out the surgical procedure. In the intervention group, patients received dextrose 50% (1g/kg) and regular insulin (1u/kg) during the preanhepatic phase. The control group received 500 mL of normal saline only during this phase. Hemodynamic parameters were monitored continuously using the Lidco system at 5-minute intervals across all three phases. Blood glucose, potassium, and arterial blood gas analyses were measured every hour. If serum potassium levels dropped below 2.5 mmol/L, or if there were any signs of hemodynamic instability or the appearance of new arrhythmias, 20 mEq of KCl was infused in 200 mL of normal saline over one hour.

### Outcome assessment

The outcomes of this study were evaluated across various perioperative and postoperative parameters to determine the impact of glucose-insulin solution administration during the preanhepatic phase of liver transplantation on the clinical and paraclinical outcomes of the patient.

Hemodynamic and Metabolic Outcomes were assessed across three transplant phases: preanhepatic, anhepatic, and reperfusion. Parameters measured included urine output, inotropic support requirement, cardiac output (CO), cardiac index (CI), peripheral vascular resistance (PVR), arterial blood gas (ABG) analysis, and bicarbonate infusion.

Laboratory Outcomes were evaluated at three points: preoperatively, postoperatively, and one week after surgery. The biomarkers assessed included alanine aminotransferase, aspartate aminotransferase, total bilirubin, brain natriuretic peptide (BNP), lactate, and creatinine.

Clinical Outcomes were measured postoperatively and included hospital length of stay, ICU stay, and duration of intubation. Additionally, overall complication rates were recorded.

### Bias

A double-blind design was employed to minimize bias in this study. Patients were unaware of the group to which they were assigned, ensuring that any preconceived expectations did not influence their responses. Similarly, the outcome assessor, who evaluated the effects of the intervention, was blinded to the group assignments and only received patient data through a coded system.

### Statistical analysis

All analyses were performed using SPSS for Windows version 27.0 (IBM, Armonk, NY, USA). Continuous data were presented as mean ± standard deviation or median with interquartile range, and categorical data were reported as frequency and percentage. Quantitative data comparison between the two groups at each time point was performed using the Independent Sample T-test or the Wilcoxon Rank-Sum Test based on data distribution. Categorical data were compared at each stage using Pearson’s Chi-Square or Fisher’s Exact Test.

The trend of continuous data was analyzed using Repeated Measures ANOVA or Friedman’s Test, and the trend of categorical data was evaluated using Generalized Estimating Equations. Repeated Mixed ANOVA (RM ANOVA), Generalized Linear Mixed Models (GLMM), and Generalized Estimating Equations were used to compare the course of the changes between the two groups. A P-value < 0.05 was considered significant.

### Ethics approval and consent to participate

All procedures followed the 1961 Declaration of Helsinki and its extensions. All participants were enrolled after describing the study and its purpose in an understandable way. Subsequently, written informed consent was obtained for participation in the research and its purpose and providing written informed consent. The study protocol was approved by the Ethics Committee of the Faculty of Medicine, Shahid Beheshti University of Medical Sciences (ID: IR.SBMU.MSP.REC.1403.076) and the clinical trial center registry (code: IRCT20240301061137N1).

## Results

**Figure-1:**
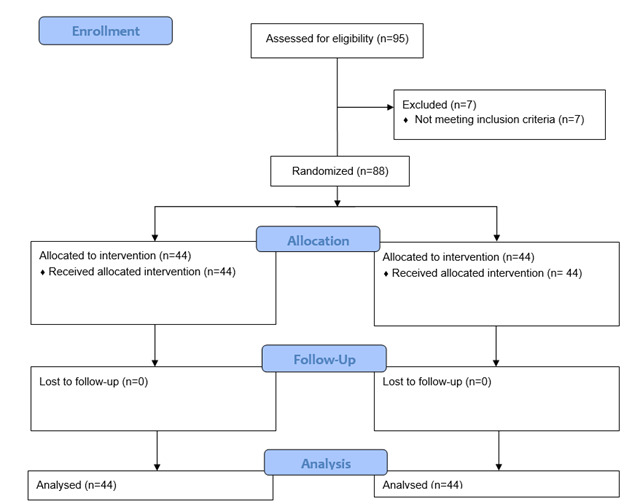
Figure 1. CONSORT flowchart of study

**Table:** Table1. Baseline characteristics of the participants in each treatment group

**Characteristic**	**Levels**		**Groups**		**P-value ^1^**
		**Overall** **(N=88)**	**Control** **(N=44)**	**Intervention** **(N=44)**	
**Age,**years		45.63±11.93	44.20± 12.87	47.05± 10.86	0.266
**Sex**	Male	45 (51.1)	24 (45.5)	21 (47.7)	0.670
	Female	43 (48.9)	20 (54.5)	23 (52.3)
**BMI,**kg/m ^2^		24.09±3.81	24.41± 3.91	23.77± 3.73	0.434
**MELD Score**		23.50 (19.00, 27.00)	22.00 (19.00, 27.00)	27.00 (19.00, 28.00)	0.142
	Chronic Hepatitis	11 (12.5)	5 (11.4)	6 (13.6)	
**Indication for Transplantation**	Cirrhosis	25 (28.4)	12 (27.3)	13 (29.5)	0.911
	HCC	5 (5.7)	2 (4.5)	3 (6.8)	
	Others	47 (53.4)	25 (56.8)	22 (50.0)	

^1^: Independent Sample T-test, Pearson’s Chi-Square Test, Wilcoxon Rank-Sum Test, Fisher’s Exact Test Quantitative data are presented as mean with standard deviation or median with interquartile range Qualitative data are presented as frequency (percentages) BMI, Body Mass Index; HCC, Hepatocellular Carcinoma

**Table:** Table2. Perioperative findings in each treatment group

**Characteristic**	**Levels**		**Groups**		**P-value ^1^**
		**Overall** **(N=88)**	**Control** **(N=44)**	**Intervention** **(N=44)**	
**Donor age,**years		41.83±12.82	41.36± 13.20	42.30± 12.57	0.735
**Liver weight,**gr		1300.00 (1000.00, 1500.00)	1300.00 (1190.00, 1500.00)	1350.00 (900.00, 1500.00)	0.170
**Intraoperative Blood Loss,**mL		1150.00 (600.00, 1500.00)	1000.00 (600.00, 1500.00)	1400.00 (525.00, 1500.00)	0.864
**Fluid intake,**mL		4000.00 (3500.00, 5000.00)	4000.00 (3250.00, 4750.00)	4500.00 (3750.00, 5000.00)	0.386
**Albumin intake,**mL		50.00 (0.00, 125.00)	50.00 (0.00, 100.00)	75.00 (0.00, 150.00)	0.363

^1^: Independent Sample T-test, Wilcoxon Rank-Sum Test Quantitative data are presented as mean with standard deviation or median with interquartile range Qualitative data are presented as frequency (percentages)

Ninety-five patients referred to the Taleghani Hospital, a referral teaching hospital in Tehran, Iran, were included in the study. Seven patients who did not meet the inclusion criteria or expired dropped out of the study. Ultimately, 88 patients remained until the end of the treatment period, as shown in Figure-1.

### General Characteristics

Eighty-eight liver transplant patients were included in the final analysis with a 1:1 ratio of control and intervention arms. The mean age of the population was 45.63±11.93 years, and 45 patients (51.1%) were female. The population’s mean body mass index (BMI) was 24.09±3.81 kg/m², and the mean Model for End-Stage Liver Disease (MELD) score was 23.91±5.14. Baseline factors, including age (P=0.266), sex (P=0.670), BMI (P=0.434), and MELD score (P=0.142), were comparable between both groups. The detailed baseline characteristics are provided in Table-1.

### Preoperative Findings

The mean age of the donors was 41.83±12.80 years, and the mean graft weight was 1288.81±328.66 grams. The mean estimated blood loss was 1115.34±533.22 mL, and the mean intraoperative fluid intake was 4187.50±995.13 mL. The mean intraoperative albumin infusion was 82.39±82.01 mL. For the perioperative parameters, no difference existed between the groups (Table-2).

## Hemodynamic and Blood Gas Results

**Table:** Table3. Patients’ outcomes and complications in each treatment group

**Characteristics**			**Groups**		**P-value ^1^**
		Overall (N=88)	Control (N=44)	Intervention (N=44)	
**Length of hospitalization,**days		13.00 (10.00, 14.00)	13.00 (10.00, 14.00)	12.50 (11.25, 13.00)	0.787
**ICU stay,**days		6.00 (5.00, 6.00)	6.00 (5.00, 6.00)	6.00 (5.00, 7.00)	0.717
**Intubation time,**hours		20.00 (16.00, 24.00)	19.00 (16.00, 24.00)	21.00 (16.00, 24.00)	0.521
**Complications**	Infection	3 (3.4)	2 (4.5)	1 (2.3)	0.616
	Portal HTN	1 (1.1)	1 (2.3)	0 (0)	

^1^: Wilcoxon Rank-Sum Test, Fisher’s Exact Test Quantitative data are presented as median with interquartile range Qualitative data are presented as frequency and percentages

### Preanhepatic Phase

During the preanhepatic period, the mean urine output was 870.40±609.90 mL, and inotropic support was needed in 35 patients (39.8%). The mean cardiac output (CO) was 6.23±1.77 L/min, with a mean peripheral vascular resistance (PVR) of 879.69±380.77 PRU and a mean cardiac index (CI) of 3.97±1.43 L/min/m². Arterial blood gas (ABG) analysis revealed a mean pH of 7.39±0.07, partial pressure of carbon dioxide (PCO₂) of 35.28±5.31 mmHg, and bicarbonate (HCO₃) of 22.33±4.26 mEq/L. Bicarbonate infusion was 59.26±37.43 mL on average. Preanhepatic phase parameters were not different between the groups (Supplementary Table-1).

### Anhepatic Phase

The anhepatic phase mean urine output was 285.91±321.23 mL. Inotropic support was required in 39 patients (88.6%) in the control group and 29 patients (65.9%) in the intervention group. The mean CO was higher in the intervention group compared to the control group (6.95±1.66 L/min vs. 5.56±2.02 L/min; P<0.001). The CI was also higher in the intervention group (3.95±0.83 vs. 3.43±1.38 L/min/m²; P=0.037), and so was the HCO₃ level (22.12±3.00 vs. 20.57±3.35 mEq/L; P=0.025). PVR demonstrated borderline significance toward lower values in the intervention group (842.25±443.72 vs. 1079.75±554.53 PRU, P=0.063). Moreover, the intervention group also required less bicarbonate infusion (56.82±39.44 vs. 103.7±52.67 mL; P=0.055) and had lower PCO₂ levels (32.26±4.87 vs. 34.45±5.08 mmHg; P=0.042). Complete anhepatic phase data are presented in Supplementary Table-2.

### Reperfusion Phase

In the reperfusion phase, the urine output was 755.34±519.33 mL on average. Inotropic support was required in 30 patients (68.2%) of the control group and 19 patients (43.2%) of the intervention group (P=0.018). Mean CO was greater in the intervention group (9.55±1.95 vs. 8.45±3.01 L/min; P=0.046), with greater CI (5.58±0.91 vs. 4.89±1.59 L/min/m²; P=0.015). PCO₂ levels were lower in the intervention group (36.03±6.25 vs. 39.06±6.22 mmHg; P=0.025) and received significantly less bicarbonate infusion (35.80±13.59 vs. 55.57±41.66 mL; P<0.001). pH and PVR values were not statistically different. Complete reperfusion phase data are presented in Supplementary Table-3.

## Laboratory Evaluation

### Preoperative laboratory findings

Preoperative laboratory results demonstrated a mean creatinine 1.00±0.61 mg/dL, ALT 135.90±286.07 U/L, AST 210.85±546.72 U/L, total bilirubin 7.93±13.79 mg/dL, BNP 374.98±791.83 pg/mL, and lactate 13.33±6.83 mmol/L. No significant difference was observed between the two groups.

## Post-operative Laboratory Results

**Table:** Table4. Assessing changes across the 3 phases of transplantation in each treatment group

**Characteristics**	**Groups**		**Time**		**P-value ^1^**	**P-value ^2^**
		Preanhepatic	Anhepatic	Reperfusion		
**Urine Output,**mL	Control	700.00 (212.50, 1425.00)	200.00 (50.00, 500.00)	600.00 (500.00, 1275.00)	<0.001	0.244
	Intervention	1250.00 (200.00, 1400.00)	150.00 (50.00, 450.00)	425.00 (262.50, 1200.00)	<0.001	
**Inotrope need**	Control	19 (43.2)	39 (88.6)	30 (68.2)	<0.001	0.141
	Intervention	16 (36.4)	29 (65.9)	19 (43.2)	<0.001	
**Cardiac Output,**L/min	Control	6.19± 1.95	5.56± 2.02	8.45± 3.01	<0.001	0.025
	Intervention	6.26± 1.59	6.95± 1.66	9.55± 1.95	<0.001	
**PVR,**PRU	Control	938.50 (712.50, 1123.50)	1010.00 (746.25, 1251.75)	700.00 (560.00, 877.50)	<0.001	0.751
	Intervention	1077.50 (416.50, 1152.75)	591.00 (436.50, 1294.50)	643.50 (363.00, 900.00)	<0.001	
**Cardiac Index,**L/min.m ^2^	Control	3.90 (2.95, 4.37)	3.35 (2.35, 4.30)	4.55 (4.00, 6.00)	<0.001	0.081
	Intervention	4.26 (3.72, 4.48)	3.85 (3.45, 4.37)	5.38 (4.92, 6.20)	<0.001	
**pH**	Control	7.40 (7.36, 7.44)	7.38 (7.35, 7.44)	7.33 (7.30, 7.40)	0.002	0.229
	Intervention	7.41 (7.37, 7.45)	7.43 (7.29, 7.45)	7.35 (7.28, 7.39)	<0.001	
**PCO2,**mmHg	Control	34.00 (31.64, 39.60)	34.50 (30.25, 37.85)	38.00 (35.00, 44.37)	<0.001	0.147
	Intervention	35.60 (31.74, 38.20)	32.76 29.17, 34.96)	35.51 (31.47, 39.67)	0.007	
**HCO3,**mEq/L	Control	21.60 (19.92, 25.37)	20.75 (18.60, 23.00)	22.00 (20.00, 23.00)	0.144	0.074
	Intervention	24.90 (17.37, 25.48)	22.25 (19.54, 24.93)	22.62 (20.67, 23.99)	0.853	
**Bicarbonate intake,**mL	Control	50.00 (50.00, 100.00)	50.00 (50.00, 100.00)	50.00 (50.00, 50.00)	<0.001	0.005
	Intervention	45.00 (25.00, 100.00)	50.00 (20.00, 100.00)	30.00 (25.00, 50.00)	0.300	

^1^: Friedman’s Test, Repeated Measure ANOVA, Generalized Estimated Equations ^2^: Repeated Mixed ANOVA, Generalized Linear Mixed Models, Generalized Estimated Equations Quantitative data are presented as mean with standard deviation or median with interquartile range Qualitative data are presented as frequency (percentages)

**Table:** Table5. Assessing laboratory changes across the study period in each treatment group

**Characteristics**	**Groups**		**Time**		**P-value ^1^**	**P-value ^2^**
		**Peri-operation**	**Post-operation**	**1-week F/U**		
**Creatinine,**mg/dL	Control	0.84 (0.675, 1.09)	0.83 (0.72, 1.18)	0.86 (0.71, 1.24)	0.910	0.923
	Intervention	0.86 (0.62, 1.23)	1.09 (0.58, 1.18)	0.84 (0.54, 1.08)	0.020	
**ALT,**IU/L	Control	46.00 (29.25, 111.00)	262.50 (59.50, 734.50)	106.50 (61.00, 261.25)	<0.001	0.485
	Intervention	25.54 (22.49, 89.69)	127.50 (37.40, 329.00)	45.74 (39.22, 211.75)	<0.001	
**AST,**IU/L	Control	72.50 (41.75, 133.50)	455.00 (103.75, 857.50)	45.00 (28.50, 104.75)	<0.001	0.211
	Intervention	31.53 (27.40, 121.23)	186.50 (61.08, 383.00)	65.50 (23.63, 95.38)	<0.001	
**Bili T,**mg/dL	Control	4.15 (1.62, 9.20)	3.64 (2.03, 9.06)	1.87 (1.16, 5.46)	<0.001	0.088
	Intervention	8.17 (1.01, 9.54)	1.49 (1.25, 8.77)	0.80 (0.55, 3.53)	<0.001	
**BNP,**mg/dL	Control	102.50 (22.15, 358.95)	119.00 (31.17, 222.67)	298.05 (52.35, 2382.30)	0.003	<0.001
	Intervention	306.73 (16.02, 469.11)	76.55 (22.36, 115.65)	35.19 (29.11, 132.62)	0.412	
**Lactate,**mg/dL	Control	12.50 (8.00, 19.00)	18.00 (11.00, 25.00)	20.50 (11.00, 26.75)	0.004	0.029
	Intervention	13.00 (7.00, 17.00)	9.00 (8.00, 20.00)	12.00 (10.00, 17.50)	0.021	

^1^: Friedman’s Test ^2^: Generalized Linear Mixed Models Quantitative data are presented as median with interquartile range

Post-operative creatinine was levels similar between the two groups (P=0.570); however, the intervention group demonstrated significantly lower ALT (P=0.038), AST (P=0.019), total bilirubin (P=0.021), BNP (P=0.002), and lactate (P<0.001) levels.

### Follow-Up Laboratory Evaluation

Upon follow-up, creatinine levels showed a borderline difference (P=0.069), whereas ALT (P=0.024), total bilirubin (P=0.002), BNP (P<0.001), and lactate (P=0.014) were lower in the intervention group. AST did not show any significant difference (P=0.152).

### Clinical Outcomes and Complications

Hospital length of stay averaged 12.33±2.19 days, with an average ICU stay of 5.81±1.02 days and intubation duration of 19.39±4.32 hours. No statistically significant differences were noted for hospital stay (P=0.787), ICU stay (P=0.717), and duration of intubation (P=0.521). Complications occurred in a total of four patients (4.5%), as three infections (3.4%), and one portal vein hypertension (1.1%). There were no differences between groups for complication rates (P=0.616) (Table-3).

### Course of Changes

Throughout the study phases, most hemodynamic and biochemical changes reached statistical significance. The HCO₃ trend was non-significant in either group and bicarbonate intake did not significantly change in the intervention group. The intervention group had better CO improvement and less bicarbonate usage than the control group. Laboratory trends demonstrated significant changes in both groups except for creatinine levels in the control group and BNP levels in the intervention group. Additionally, changes in the BNP and lactate levels significantly differed between the two groups. Perioperative, post-operative, and follow-up changes are presented in Tables-4and5.

## Discussion

This randomized clinical trial evaluated the impact of glucose-insulin infusion during the preanhepatic stage of liver transplantation on paraclinical and clinical outcomes. The results indicated that infusion of glucose-insulin solution during the perioperative period resulted in good hemodynamic and biochemical profiles in later stages of transplantation, as supported by good postoperative laboratory results such as liver enzymes level, B-type natriuretic peptide (BNP), and lactate level.

Similar to the study by Cywes et al. [15], which demonstrated that intraportal glucose-insulin infusion in liver donors increased hepatic glycogen content and reduced postoperative aspartate aminotransferase (AST) levels, particularly with prolonged anoxic rewarming times, the current study found improved metabolic outcomes, including significantly lower postoperative AST (P=0.019), alanine aminotransferase (ALT) (P=0.038), and bilirubin (P=0.021) levels in the intervention group receiving dextrose-insulin infusion. These results suggest a protective effect on hepatocyte function, consistent with Cywes et al.’s observation of enhanced glycogenation and reduced liver injury. However, unlike the study by Gedik et al. [13], which reported safer blood glucose levels with dextrose alone compared to dextrose plus insulin due to hyperglycemic risks during the neohepatic phase, the current trial observed no significant hyperglycemia or hypoglycemia, likely due to the double-blinded, controlled administration of dextrose (1g/kg) and insulin (1u/kg). Compared to Kang et al. [9], who found that Portland intensive insulin therapy (PoIIT) reduced intraoperative hyperglycemia and postoperative complications like major infections and biliary stricture, the current study did not observe differences in complication rates or hospital stay, possibly due to differences in insulin dosing protocols or patient populations. Similarly, while Omiya et al. [12] and Hassanain et al. [10] reported reduced surgical site infections and improved liver function with hyperinsulinemic-normoglycemic clamp (HNC) protocols, the current study’s focus on preanhepatic infusion uniquely demonstrated enhanced hemodynamic stability, with higher cardiac output (6.95±1.66 vs. 5.56±2.02 L/min, P<0.001) and lower inotropic requirements (65.9% vs. 88.6%, p=0.018) during the anhepatic phase. These findings suggest that preanhepatic dextrose-insulin infusion not only supports metabolic stability, as seen in Sato et al. [14], but also improves intraoperative hemodynamics, potentially by optimizing energy availability and reducing metabolic stress, without increasing adverse events.

Also, in previous studies, insulin-glucose infusion has been demonstrated to improve hemodynamic stability and myocardial function. For example, Ellenberger et al. showed that glucose-insulin-potassium infusion in patients undergoing cardiac surgery maintained left ventricular systolic function and decreased inotropic needs [16]. Likewise, Shim et al. illustrated better myocardial performance and hemodynamic stability in patients with acute coronary syndrome undergoing coronary artery bypass graft (CABG) surgery with insulin treatment [17]. Based on these results, our trial showed better hemodynamic parameters in the intervention group with increased cardiac output and cardiac index and reduced requirements for inotropic support. This finding could be attributed to the inotropic action of insulin, which has been long established to increase myocardial metabolism through an improved supply of adenosine-3-phosphate (ATP) substrate [18][19]. Furthermore, significantly lower BNP levels in the intervention group also point toward the potential beneficial influence of insulin therapy on minimizing cardiac stress [20].

Although not statistically significant, the trend for reduced peripheral vascular resistance (PVR) in the intervention group can be a fair representation of insulin’s vasodilatory effect [21]. Since no significant difference was observed between the two groups, it would suggest that vasodilation was not the prevailing mechanism for hemodynamic improvement. Taken together, these results offer a plausible explanation for the reduction in inotropic demand for the intervention group. Nevertheless, the observed disparities in cardiac index responses and inotropic requirements between the two groups remained of borderline significance, calling for further investigation.

Contrary to hemodynamic variables, renal function remained unchanged between groups, as attested to by similar urine output and creatinine levels throughout the transplantation periods. The results imply that organ perfusion and kidney function were maintained irrespective of intervention [22][23]. Since renal function is determined by systemic vascular resistance and renal blood flow, this absence of appreciable differences could be due to similar PVR and fluid and albumin intake in both groups [5].

In addition, glucose-insulin therapy had a metabolic impact, as evidenced by decreased partial pressure of carbon dioxide (PCO₂) and increased bicarbonate (HCO₃⁻) in the intervention group, indicating better acid-base homeostasis. Insulin has a key role in optimizing glucose metabolism and minimizing metabolic CO₂ production, which can account for our patients’ better acid-base status [24], which could be mediated through improved cardiorespiratory responses to hemodynamic fluctuations during liver transplantation [25][26][27]. This effect was most pronounced during the reperfusion period, with significantly less bicarbonate requirement in the intervention group. On the contrary, no significant difference was observed between the two groups regarding bicarbonate requirement in the anhepatic phase, which could be attributed to the high metabolic demands and increased lactate production during this phase [28][29]. Since liver ischemia during the anhepatic period causes severe metabolic stress, the fact that there was not a noticeable decrease in bicarbonate requirement in this period shows that the metabolic effects of insulin may be more pronounced in subsequent periods of transplantation [30][31]. This effect could be due to reduced metabolic stress and improved cellular function in this group [32].

The hepatoprotective action of insulin-glucose infusion was manifested as significantly lower postoperative ALT and AST levels in the intervention group. These results are consistent with earlier observations that insulin has a protective effect on hepatocytes by its anti-apoptotic action, apart from minimizing metabolic stress and streamlining glucose metabolism. This raises the potential for the use of insulin in preventing ischemia-reperfusion injury in liver transplantation [33][34].

Despite these metabolic and biochemical advantages, the intervention did not lead to better clinical outcomes. There were no significant differences in perioperative outcomes, such as hospital length of stay, ICU stay, or mechanical ventilation duration. These results indicate that although insulin-glucose infusion can exert positive physiologic effects, these effects alone are not enough to produce significant clinical advantages, probably because postoperative recovery is multifactorial and will be affected by baseline patient status, complexity of surgery, hemodynamic stability, and anesthetic management [35].

Additionally, the incidence of postoperative complications did not vary significantly among groups. The most common complication was infection, which happened in three patients, whereas one instance of portal hypertension was registered in the control group. However, because of the small sample size, conclusions regarding the effect of glucose-insulin infusion on postoperative morbidity cannot be established.

This study is one of the few studies that have evaluated the safety and efficacy of glucose-insulin infusion in liver transplantation, but it has several limitations. The single-center nature and the relatively small number of patients limit the generalizability of the results. Longer follow-up durations may also be required to determine the potential long-term advantages of this therapy.

In conclusion, our results indicate that preanhepatic glucose-insulin infusion in liver transplantation can exert metabolic and hemodynamic advantages of improved myocardial performance, better acid-base status, and less hepatocellular damage; however, these physiological enhancements were not linked to shorter hospital stays, ICU stays, and mechanical ventilation. More extensive, multicenter studies with longer follow-ups are needed to further define the clinical significance of this therapy in liver transplantation outcomes.

## Conflict of Interest

The authors have no relevant financial or non-financial interests to disclose.
